# The New Orleans Medical Journal

**Published:** 1844-12

**Authors:** 


### BIBLIOGRAPHICAL NOTICES.

*The New Orleans Medical Journal.*—We have received (in exchange) the 2d and 3d numbers of this periodical. Its first number was issued in May last, under the editorial conduct of Erasmus D. Fenner, M.D., and A. Hester, M.D., one of the Physicians to the New Orleans Charity Hospital. Each number contains 128 octavo pages, and is issued at the beginning of every other month, at a cost of $5,00 per annum. Much merit is due to the projectors of this enterprise and many thanks from the profession generally. We have long felt the want of some source, upon which we could rely for information, respecting the dreaded diseases of the sourthern part of the United States. This is supplied by the valuable publication before us. We have perused its pages with interest and profit, and hope its circulation may be wide and rapid, both for the good of the profession, and as a just reward of the merits of the undertaking. We hope that we may be favored with the 1st No., which we have not yet received.

